# The impact of endometrioma and laparoscopic cystectomy on serum anti-Müllerian hormone levels

**DOI:** 10.1186/1477-7827-9-80

**Published:** 2011-06-09

**Authors:** Yuh-Ming Hwu, Frank Shao-Ying Wu, Sheng-Hsiang Li, Fang-Ju Sun, Ming-Huei Lin, Robert Kuo-Kuang Lee

**Affiliations:** 1 Department of Obstetrics and Gynecology, Mackay Memorial Hospital, Taipei, Taiwan; 2 Mackay Medicine, Nursing and Management College, Taipei, Taiwan; 3 Department of Medical Research, Mackay Memorial Hospital, Taipei, Taiwan

## Abstract

**Background:**

Serum anti-Müllerian hormone (AMH) had been proposed as a marker of ovarian reserve. The aim of this study was to evaluate the impact of endometrioma and laparoscopic cystectomy on ovarian reserve as measured by serum AMH levels.

**Methods:**

A total of 1,642 patients were recruited in this retrospective analysis. Control group (group 1) included 1,323 infertility patients without endometrioma. Endometrioma group (group 2) included 141 patients with ovarian endometrioma. Previous cystectomy group (group 3) included 147 patients who underwent unilateral or bilateral laparoscopic cystectomy due to ovarian endometrioma more than 6 months before enrollment. Current cystectomy group (group 4) included 31 patients who underwent cystectomy during study period. Serum anti-müllerian hormone (AMH) levels were measured upon enrollment with all patients. For patients in group 4, AMH levels were measured before and 3 months after cystectomy.

**Results:**

Mean AMH level of patients in control group was significantly higher than that of endometrioma group or previous cystectomy group in each age subgroup, while the mean serum AMH level of the endometrioma group was also significantly higher than that of the previous cystectomy group in each age subgroup. The mean AMH level was significantly lower in patients with previous bilateral cystectomy compared to that of patients with unilateral cystectomy. The mean serum AMH level was also significantly lower in patients with bilateral endometrioma compared to that of patients with unilateral endometrioma. In group 4, mean AMH level significantly decreased from 3.95 +/- 0.42 preoperation to 2.01 +/- 0.21 ng/ml at 3-month postoperation.

**Conclusions:**

Both ovarian endometrioma and cystectomy are associated with a significant reduction on ovarian reserve. Bilateral endometrioma exerts a more profound negative impact on ovarian reserve than unilateral endometrioma, regardless of either conservative or surgical intervention.

## Background

Endometrioma is one of the most commonly encountered diagnoses in ovarian surgery and may be present in up to 17-44% of patients with endometriosis [1-3]. Ovarian endometriomas are usually associated with the symptoms of dysmenorrhea, chronic pelvis pain, dyspareunia, and infertility. Previous studies have demonstrated that endometriomas can negatively affect the rate of spontaneous ovulation [4], as well as reducing the amount of follicular number and activity in the adjacent ovarian tissues [5]. Based on articles review, the current 2008 ESHRE guidelines on endometriosis recommended that laparoscopic ovarian cystectomy is advised if an ovarian endometrioma ≥ 4 cm in diameter is present and hereby to confirm the diagnosis histologically, improve access to follicles, and possibly improve ovarian response [6-8]. However, some studies reported that ovarian reserve was damaged after excision of endometriomas [4,9-11] and therefore, ESHRE guidelines also recommended that these patients should be counseled on the risk of reduced ovarian function after surgery. With the difficulties to evaluate the ovarian reserve, most of the previous studies used ovarian responses to gonadotropins as a marker to assess the ovarian reserve after endometrioma cystectomy [4,9-11].

In the past two decades, serum anti-Müllerian hormone (AMH), day 3 FSH, E2, and inhibin B levels have been proposed as markers of ovarian reserve [12-14]. However, inhibin B, FSH, and E2 levels are all involved in the pituitary-ovary axis negative feedback [15] that great variations are observed during menstrual cycle. In contrast, serum AMH levels were reported to be stable throughout the menstrual cycle [13]. La marca et al. recommended that AMH was a superior marker for predicting ovarian response over either age, FSH, or inhibin B [14]. Therefore, AMH may also be a very informative marker regarding the degree of ovarian reserve damage due to endometrioma or ovarian cystectomy. However, very few studies have assessed the damage on ovarian reserve induced by ovarian endometrioma or cystectomy using serum AMH levels. The aim of this study was to evaluate the impact of endometrioma and laparoscopic cystectomy on ovarian reserve as measured by serum AMH levels. In addition, the differences in ovarian reserve impairment between unilateral and bilateral endometriomas and cystectomy were also investigated.

## Methods

### Patients

A total of 1,642 subjects were recruited in this retrospective study. Enrolled were infertility patients with or without ovarian endometrioma between the age of 22 to 46-year-old visiting MacKay Memorial Hospital between January 2007 and March 2010. The study protocol was approved by the Institutional Review Board (IRB) of Mackay Memorial Hospital in Taipei, Taiwan. Patients with subfertility problem were recruited after evaluation for infertility, including hysterosalpingography, endocrine evaluation, and ultrasonography for female patients, and semen analysis for their male partners.

Serum levels of endocrines including total testosterone, prolactin, 17-hydroxyprogesterone (17-HP), dehydroepiandrosterone sulfate (DHEAS), free thyroxine (T4), thyroid-stimulating hormone (TSH), and AMH were obtained in the early follicular phase of menstrual cycle. The exclusion criteria were as follows: patients with [1] polycystic ovarian syndrome according to the Rotterdam criteria [16], [2] ovarian malignant diseases, [3] intake of hormonal medications within 3 months before enrollment, such as oral contraceptive pills, GnRH analogue or danazol treatment, [4] a body mass index (BMI) higher than 30 kg/m^2^, [5] evidence of endocrine disorders such as hyperprolactinemia, congenital adrenal hyperplasia, Cushing's syndrome, or adrenal gland tumor.

Subjects were divided into four groups according to the presence or status of endometrioma (Table 1). Group 1 (control group) (n = 1,323): patients were recruited from outpatient department due to subfertility problem. No ovarian endometrioma were found using ultrasonography in the patients of this group. Serum AMH levels were obtained upon enrollment. Group 2 (endometrioma group) (n = 141): Patients with unilateral or bilateral ovarian endometrioma with a diameter of at least 3 cm was diagnosed by clinical and ultrasonography findings with no surgical intervention during enrollment. Group 3 (endometrioma with previous cystectomy group) (n = 147): Patients who underwent unilateral or bilateral laparoscopic cystectomy due to ovarian endometrioma more than 6 months before enrollment were recruited. Serum AMH levels were obtained at time of enrollment. Serum AMH levels were not measured before the previous laparoscopic cystectomy. Group 4 (current cystectomy group) (n = 31): Unilateral ovarian endometrioma with a diameter of at least 3 cm was diagnosed upon enrollment and laparoscopic unilateral cystectomy was performed aftermath. Ovarian endometrioma was confirmed by histology specimen obtained from laparoscopic cystectomy. Serum AMH levels were obtained both before and 3 months after laparoscopic cystectomy (Table 1). Patients with bilateral endometrioma undergoing bilateral cystectomy were excluded in this current cystectomy group.

**Table 1:** Clinical characteristics of the patients in each group

	Number of patients	Age (Mean ± SD) (Range)	Clinical Characteristics	Laparoscopic Cystectomy	Time of serum AMH collection
Control Group (Group 1)	1,323	34.09 ± 4.34 (22-46)	Tubal factor Male factor Unexplained infertility	nil	At time of enrollment
Endometrioma Group (Group 2)	141	33.27 ± 4.09 (23-44)	Endometrioma > 3 cm diagnosed by ultrasound and clinical diagnosis	nil	At time of enrollment
Previous Cystectomy Group (Group 3)	147	33.88 ± 4.29 (22-47)	Previous laparoscopic cystectomy due to ovarian endometrioma	Cystectomy performed more than 6 months before enrollment	At time of enrollment
Current Cystectomy Group (Group 4)	31	31.14 ± 3.96 (22-39)	Unilateral endometrioma with laparoscopic cystectomy performed after enrollment	Unilateral cystectomy was performed after enrollment	Before cystectomy and 3 months after cystectomy

### Serum AMH measurements

Serum AMH levels were measured in the early follicular phase of menstrual cycle. For groups 1, 2, and 3, serum AMH levels were obtained at outpatient department upon enrollment. For group 4, AMH levels were measured preoperatively upon enrollment and 3 months postoperatively. AMH levels were measured using a commercial enzyme-linked immunosorbent assay kit (ELISA, Diagnostic Systems Laboratories, Webster, TX) and the lowest detectable level of AMH distinguishable from zero was 0.006 ng/ml. The intra-assay and inter-assay coefficients of variation were 4.6% and 8.0% respectively.

### Diagnosis of ovarian endometrioma

All recruited patients underwent transvaginal ultrasonography evaluation using a Toshiba Nemio SSA-550A System. The color and power Doppler imaging evaluation was also used upon need. According to Guerriero's study [17] and other previous reports, criteria for ultrasound diagnosis of endometrioma were: [1] cystic structure with homogenous low-level internal echoes without papillary proliferations associated with poor vascularization, and [2] cystic structure with homogenous low-level internal echoes with an echogenic portion in which no flow was detected [17,18]. Follow-up ultrasound examination was performed 1-2 months aftermath to exclude spontaneously resolving hemorrhagic cyst.

### Laparoscopic cystectomy techniques

All laparoscopic cystectomy operations were performed under general anesthesia during the early or middle follicular phase of menstrual cycle. The cleavage plane between the cyst wall and the normal ovarian tissue was identified after the cyst wall was incised with monopolar scissors. Then, the cyst wall was completely stripped off from the normal ovarian tissue by traction and opposite traction with two grasping forceps. Hemostasis was performed with bipolar forceps electrocoagulation. All endometrioma specimens obtained from operation were submitted for pathology examination.

### Statistical analysis

Data were expressed as mean ± standard error of mean (SEM). One way analysis of variance (ANOVA) followed by the post hoc test was employed to compare the significance of differences in serum AMH levels between the control, endometrioma and cystectomy groups. The relationships between the changes in serum AMH levels and patient's age were analyzed using bivariate correlation analysis with Pearson coefficient. The relationships were presented graphically by regression curve estimation using Medcalc and SigmaPlot software.

Student's *t*-test was used to compare the differences in AMH levels between unilateral and bilateral endometriomas and cystectomy. Paired *t*-test was used to compare the differences between the sampling points (preoperative and 3 months postoperative) for the changes in AMH levels of group 4. The statistical software package SPSS version 12.0 (SPSS Inc., Chicago, IL) and SigmaPlot (Systat Software Inc., Chicago, IL) were used. All results were considered statistically significant at P < 0.05.

## Results

### The effect of endometrioma and previous cystectomy on serum AMH levels

**Figure 1:**
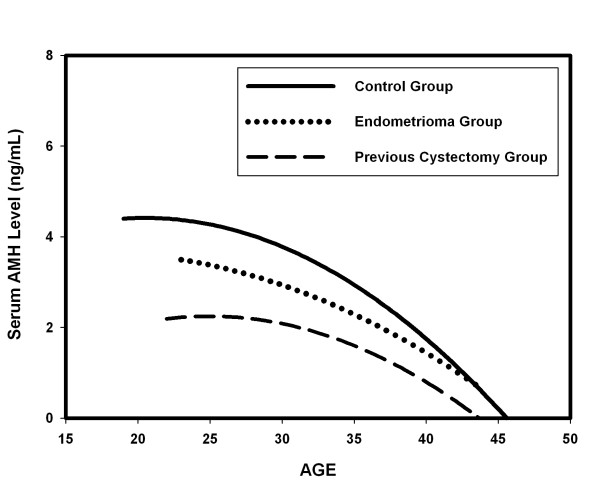
**The impact of endometrioma and previous cystectomy on serum AMH levels in relation to age**. The estimated curves of serum AMH levels in relation to age represent the patients in control group, endometrioma group and previous cystectomy group respectively. Serum AMH level was significantly negatively correlated with increasing age in all groups.

A total of 1,642 patients were included in this study, with a mean age of 33.91 years at their initial visit (range: 22-46 years), and their clinical characteristics are shown in Table 1. The serum levels of AMH in relation to age in the control group, endometrioma group, and previous cystectomy group are all shown in Figure 1. In control group, serum AMH level was significantly negatively correlated with increasing age of the patients (*r *= -0.421, P < 0.001) (Figure 1). Similar to control group, serum AMH levels were also significantly negatively correlated with increasing age in both endometrioma group (*r *= -0.364, P < 0.001) and previous cystectomy group (*r *= -0.266, P < 0.01) (Figure 1). The mean ages of patients in each group are shown in Table 1. No significant difference was found regarding the mean ages of patients between the groups (Table 1).

**Figure 2:**
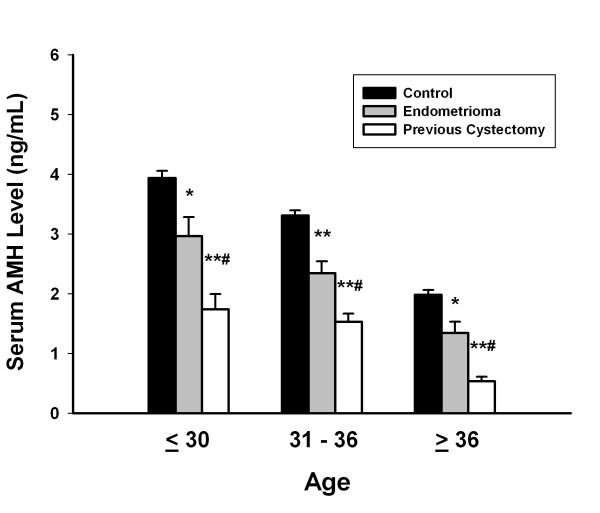
**The effect of endometrioma and previous cystectomy on serum AMH levels in each age subgroup**. The mean serum AMH level of patients in the control group was significantly higher than that of the patients in the endometrioma group or previous cystectomy group in each age subgroup. Data are shown as the mean ± SEM. One way analysis of variance (ANOVA) followed by the post hoc test was employed to compare the significance of differences. *P < 0.001, control group vs. cystectomy group. ** P < 0.05, control group vs. endometrioma group. # P < 0.05, endometrioma group vs. cystectomy group. ## P < 0.001, control group vs. endometrioma group.

To assess the effect of endometrioma and previous cystectomy on serum AMH levels, one way analysis of variance (ANOVA) was applied and it presented significant differences in the mean serum AMH levels among the control group, endometrioma group, and previous cystectomy group in each age subgroup (Table 2) (Figure 2). Post hoc test showed that the mean serum AMH level of patients in control group was significantly higher than that of the endometrioma group or previous cystectomy group in each age subgroup (Table 2) (Figure 2). The mean serum AMH level of the endometrioma group was also significantly higher than that of the previous cystectomy group in each age subgroup (Table 2) (Figure 2).

**Table 2:** Comparison of mean serum AMH levels (ng/mL) between the groups according to age

Age	Control Group	Endometrioma Group	Cystectomy Group	P Value (ANOVA test)
≦30	3.94 ± 0.12	2.97 ± 0.31 ^b^	1.74 ± 0.25 ^a, c^	<0.001
31-35	3.31 ± 0.08	2.34 ± 0.19 ^d^	1.53 ± 0.14 ^a, c^	<0.001
≧36	1.98 ± 0.08	1.35 ± 0.19 ^b^	0.53 ± 0.07^a, c^	<0.001

Values are mean ± SEM

Post-hoc test:

a. P < 0.001;control group vs. cystectomy group

b. P < 0.05; control group vs. endometrioma group

c. P < 0.05; endometrioma group vs. cystectomy group

d. P < 0.001; control group vs. endometrioma group

### The impact of unilateral or bilateral endometrioma on serum AMH levels

**Figure 3:**
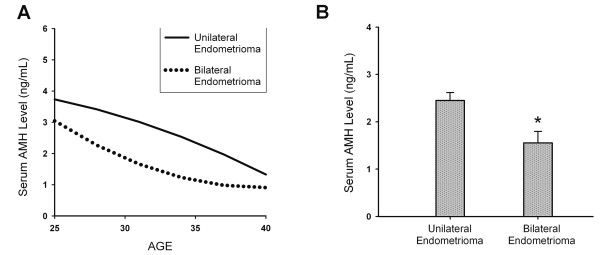
**The impact of unilateral or bilateral endometrioma on serum AMH levels**. (A) The estimated curves of serum AMH levels in relation to age for patients with unilateral endometrioma and bilateral endometriomas. (B) The mean serum AMH level was statistically significantly lower in patients with bilateral endometrioma compared to that in patients with unilateral endometrioma. Data are shown as the mean ± SEM (*P < 0.05).

To evaluate the impact of unilateral or bilateral endometrioma on serum AMH levels in endometrioma group (group 2) (n = 141), the mean serum AMH level of patients with bilateral endometrioma (n = 32) was compared with that of patients with unilateral endometrioma (n = 109). Figure 3 shows the mean serum AMH level was significantly lower in patients with bilateral endometrioma compared to that of patients with unilateral endometrioma (1.56 ± 0.24 (SEM) vs. 2.45 ± 0.17 ng/ml, P < 0.05) (Figure 3). The mean ages of patients with unilateral and bilateral endometrioma were 33.4 ± 4.1 (SD) and 32.9 ± 4.2 respectively. No significant difference was found regarding the mean ages of patients between the unilateral and bilateral endometrioma groups. Notably, four women younger than 38 years (range: 33-37 years) in endometrioma group had serum AMH levels lower than 0.5 ng/ml. One woman was diagnosed with a 5-cm diameter endometrioma in her left ovary. The other three women had bilateral endometriomas larger than 4 cm in diameter.

### The impact of unilateral or bilateral cystectomy on serum AMH levels

**Figure 4:**
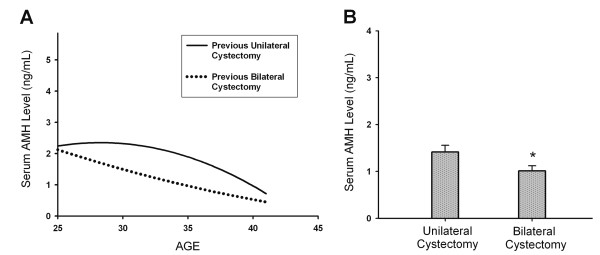
**The impact of unilateral or bilateral cystectomy on serum AMH levels**. (A) The estimated curves of serum AMH levels in relation to age for patients with previous unilateral cystectomy and bilateral cystectomy group. (B) The mean serum AMH level was statistically significantly lower in patients with previous bilateral cystectomy than those with unilateral cystectomy. Data are shown as the mean ± SEM. (*P < 0.05).

To compare the effect of unilateral and bilateral cystectomy on serum AMH levels in previous cystectomy group (group 3), the mean serum AMH level in patients with bilateral cystectomy (n = 66) was compared to that in patients with unilateral cystectomy (n = 81). Figure 4 shows the mean serum AMH level was significantly lower in patients treated with bilateral cystectomy than in patients treated with unilateral cystectomy (1.01 ± 0.11 vs.1.48 ± 0.14 ng/ml, P < 0.05) (Figure 4). The mean ages of patients with unilateral and bilateral previous cystectomy were 33. 6 ± 4.1 (SD) and 34.2 ± 4.5 respectively. No significant difference was found regarding the mean ages of patients between the unilateral and bilateral previous cystectomy groups. Among the 147 women in previous cystectomy group, two women (1.4%) younger than 38 years had ovarian failure 1 year after bilateral cystectomy. Another eight women (5.4%) younger than 38 years (range: 28-37 years) with severely impaired ovarian reserve (AMH <0.05 ng/ml, day 3 FSH >15 mIU/ml) were identified. Seven of these eight women underwent bilateral cystectomy. Another woman underwent unilateral cystectomy.

### The changes between the preoperative and 3-month postoperative serum AMH levels

**Figure 5:**
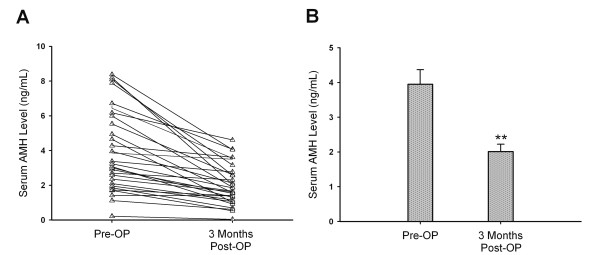
**The impact of cystectomy on serum AMH levels 3 months after operation**. (A) Changes in serum AMH levels before and 3 months after laparoscopic unilateral cystectomy in 31 patients with ovarian endometrioma. (B) The mean serum AMH level significantly decreased 3 months after laparoscopic cystectomy in 31 patients with ovarian endometrioma. Data are shown as the mean ± SEM. (**P < 0.01).

To study the extent of cystectomy damage on ovarian reserve, the preoperative and 3-month postoperative serum AMH levels were obtained from 31 patients who underwent cystectomy due to endometrioma (current cystectomy group) (group 4). Figure 5 shows the mean serum level significantly decreased from 3.95 ± 0.42 ng/ml (SEM) before cystectomy to 2.01 ± 0.21 ng/ml 3-month postoperation (P < 0.01) (Figure 5).

## Discussion

In this study, the results demonstrated that mean serum AMH level in patients with endometrioma was significantly lower than that of patients in the control group. Additionally, the mean serum AMH level in patients with previous cystectomy was also significantly lower than patients with endometrioma (without previous cystectomy) (Figures 1 and 2) (Table 2). Based on our results, we suggest that ovarian endometrioma *per se *is associated with reduced ovarian reserve, and laparoscopic cystectomy can further exerted significant damage on ovarian reserve. Meanwhile, bilateral endometriomas had a more profound impact on serum AMH levels than unilateral endometriomas, regardless of either surgical cystectomy or conservative management (Figures 3, 4, 5).

The effect of endometrioma-associated damage on ovarian reserve is very difficult to assess and quantify. In the previous studies, the damaging effect of endometrioma *per se *on ovarian reserve was assessed by histological study [5], antral follicle count [19], ovulation rate [4], or responsiveness to gonadotropins as the surrogate marker [11,20]. A meta-analysis concluded that ovarian endometrioma was associated with significantly reduced number of retrieved oocytes and developing follicles after ovarian stimulation [21]. In our study, we used serum AMH levels to assess the ovarian reserve. The mean serum AMH level of the 141 patients with endometrioma (group 2) was significantly lower compared to the 1,323 patients without endometrioma (control group) (Figures 1, 2, Table 2). Shebl et al. reported that AMH levels were significantly lower in patients with severe endometriosis than in the control group [22]. In agreement with Shebl's reports, we also found that patients with ovarian endometriomas had significantly lower serum AMH levels compared with patients without endometriomas. These results are compatible with those of the previous reports that showed the presence of endometrioma *per se *may damage the ovarian reserve measured by antral follicle count or responsiveness to gonadotropins [11,19,21].

To illustrate the difference in the adverse effect on ovarian reserve between unilateral and bilateral endometriomas, our results showed mean serum AMH level was significantly lower in patients with bilateral endometriomas than those with unilateral endometriomas (Figure 3). Based on the results in this study and previous reports, we suggest that the presence of endometrioma *per se *exerts damage on ovarian reserve. Moreover, bilateral endometriomas exert greater damage on ovarian reserve than unilateral endometriomas.

The mechanism of endometrioma inducing ovarian reserve damage is still elusive. Maneschi et al. reported that endometrioma was associated with microscopic alterations of the follicular and vascular patterns [5]. The number of follicles was reduced and vascular network was impaired. Maneschi et al. concluded that the ovarian cortical tissue alterations could be related to either the inflammatory response to the endometriosis implants or to the toxic nature of the cystic fluid [5]. Meanwhile, Fauvet et al. discovered an increased pro-apoptotic protein expression (bax and p21) in endometriomas compared with benign ovarian tumors [23]. Future study is needed to elucidate the mechanisms of ovarian reserve damage induced by endometrioma.

The real amount of surgery-mediated ovarian reserve damage can not be measured directly. In the previous reports, ovarian responsiveness to gonadotropin hyperstimulation, ovarian volume, and antral follicle count (AFC) have been used as the marker for assessing ovarian reserve damage [10,21,24-26]. Nonetheless, AFC by transvaginal ultrasound can not be used in women without sexual experience. In the literature, ovarian responsiveness is the most often used marker of ovarian reserve. However, there is controversy about the damaging effect of cystectomy on ovarian responsiveness to hyperstimulation. Some studies have found a reduced responsiveness to hyperstimulation after cystectomy [20,25-31] and most of them concluded that laparoscopic endometrioma cystectomy contributes to the reduction of ovarian reserve. In the current study, serum AMH level was used to replace ovarian responsiveness as the marker of ovarian reserve damage induced by cystectomy. In the previous reports, only a few studies have evaluated the ovarian reserve damage using serum AMH levels in women undergoing endometrioma cystectomy [32-34]. Tsolakidis et al. reported that the mean serum AMH level was significantly reduced 6 months after surgery [32]. Chang et al. also observed a significant decrease in serum AMH levels 3 months after laparoscopic cystectomy [33]. Findings of the current study are in agreement with these reports [32,33] that it showed a significant reduction in serum AMH levels in 31 patients 3 months after cystectomy (Figure 5).

During cystectomy, it is sometimes difficult to identify and separate the cleavage plane between the cyst wall and adjacent ovarian cortex tissue due to fibrotic adhesion. Cystectomy using the stripping technique usually leads to removal of normal primary follicles and damage of ovarian reserve [35,36]. Furthermore, bipolar coagulation at seriously bleeding sites close to ovarian hilus also leads to destruction of the ovarian blood supply and reduced ovarian reserve [24,37]. Excessive bipolar coagulation and inadvertent removal of normal ovarian tissues adhesive to endometrioma cyst wall may contribute to lowered AMH levels after cystectomy in this study (Figure 5).

The current study also discovered that patients undergoing bilateral cystectomy for endometrioma had significantly lower AMH levels compared with patients receiving unilateral cystectomy (Figure 4). Comparable with our results, previous studies also reported that a mean number of retrieved oocytes was significantly reduced and IVF outcome was significantly impaired in patients who have undergone bilateral cystectomy than unilateral cystectomy [9,10,30]. Busacca et al. reported that patients who operated for bilateral endometrioma had a prevalence of 2.4% ovarian failure immediately after surgery [9]. Similar to previous reports [9,10], we found that two out of 144 women (1.4%) had ovarian failure 1 year after bilateral cystectomy. Another eight out of 144 women (5.4%) younger than 38 years had severe ovarian reserve damage after cystectomy. These eight women had very poor fertility outcomes and could experience ovarian failure in the near future. In the endometrioma group, we identified four women with endometriomas younger than 38 years who had very low serum AMH levels (<0.5 ng/ml). These four women may encounter a high risk of impending ovarian failure after cystectomy. To reduce such risk, it is imperative to assess the ovarian reserve before cystectomy in women with ovarian endometrioma. As women with ovarian endometrioma and a very low serum AMH level are expected to be at high risk of impending ovarian failure after cystectomy, IVF treatment before cystectomy may offer better pregnancy outcome for them.

## Conclusions

In conclusion, the results of this study demonstrated the following observations. First, ovarian endometrioma is associated with a reduced ovarian reserve measured by serum AMH levels. Second, laparoscopic cystectomy further exerted a significant negative impact on ovarian reserve measured by serum AMH levels in both short and long term observations. Third, bilateral endometriomas had a more profound impact on ovarian reserve than unilateral endometriomas did, regardless of either conservative or surgical intervention.

For counseling of patients with ovarian endometrioma, it is imperative to identify patients with poor ovarian reserve before surgery. Assessment of ovarian reserve by serum AMH levels before cystectomy may help to prevent ovarian failure after cystectomy. The results of this study suggest that serum AMH level should be considered as a clinical routine test in patients with ovarian endometrioma before cystectomy counseling. Further large scale well-designed clinical trials are in demand to confirm this suggestion.

## Competing interests

The authors declare that they have no competing interests.

## Authors' contributions

YMH participated in the design of the study and drafted the manuscript. FSYW carried out the data collection, performed the statistical analysis. RKKL supervised the analysis. SHL and FJS carried out the data collection and statistical analysis. MHL helped in the study coordination and the data collection. All authors read and approved the final manuscript.
